# In-Vitro Subtype-Specific Modulation of HIV-1 Trans-Activator of Transcription (Tat) on RNAi Silencing Suppressor Activity and Cell Death

**DOI:** 10.3390/v11110976

**Published:** 2019-10-23

**Authors:** Larance Ronsard, Ashraf S. Yousif, Janani Ramesh, N. Sumi, Matthew Gorman, Vishnampettai G. Ramachandran, Akhil C. Banerjea

**Affiliations:** 1 Laboratory of Virology, National Institute of Immunology, New Delhi 110067, India; 2 Department of Microbiology, University College of Medical Sciences and Guru Teg Bahadur Hospital, Delhi 110095, India; rama_88@yahoo.com; 3 Ragon Institute of MGH, MIT and Harvard University, 400 Technology Square, Cambridge, MA 02139, USA; AHAMADELNEEL@mgh.harvard.edu (A.S.Y.); mgorman8@mgh.harvard.edu (M.G.); 4 Renal Division, Brigham and Women’s Hospital, Harvard Medical School, Boston, MA 02115, USA; rjjananiramesh95@gmail.com; 5 Department of Medical Biochemistry, Dr. A. L. M. Postgraduate Institute of Biomedical Sciences, University of Madras, Chennai, Tamil Nadu 600113, India; 6 Endocrinology & Toxicology Lab, Department of Zoology, University of Calicut, Kerala 673635, India; sumikrishna24@gmail.com

**Keywords:** human immunodeficiency virus (HIV)-1, trans-activator of transcription (Tat) gene, acquired immunodeficiency syndrome (AIDS), RNA interference (RNAi) silencing suppressor (RSS) activity and cell death

## Abstract

Human immunodeficiency virus (HIV) is a global health concern affecting millions of individuals with a wide variety of currently circulating subtypes affecting various regions of the globe. HIV relies on multiple regulatory proteins to modify the host cell to promote replication in infected T cells, and these regulatory proteins can have subtle phenotypic differences between subtypes. One of these proteins, HIV-1 Trans-Activator of Transcription (Tat), is capable of RNA interference (RNAi) Silencing Suppressor (RSS) activity and induction of cell death in T cells. However, the subtype-specific RSS activity and induction of cell death have not been explored. We investigated the ability of Tat subtypes and variants to induce RSS activity and cell death. TatB, from HIV-1 subtype B, was found to be a potent RSS activator by 40% whereas TatC, from HIV-1 subtype C, showed 15% RSS activity while subtype TatC variants exhibited varying levels. A high level of cell death (50–53%) was induced by subtype TatB when compared to subtype TatC (25–28%) and varying levels were observed with subtype TatC variants. These differential activities could be due to variations in the functional domains of Tat. These observations further our understanding of subtype-specific augmentation of Tat in HIV-1 replication and pathogenesis.

## 1. Introduction

Among all the genetic human immunodeficiency virus (HIV) subtypes that exist in the world, subtype C is responsible for ~50% of HIV-1 infections globally which included parts of Africa and Asia regions namely China, India etc., whereas in North America and Europe, the majority of HIV infections are caused by subtype B [1,2]. HIV-1 has the ability to rapidly mutate in infected individuals [3] and take advantage of host genes like C-C chemokine receptor type 5 (CCR5) [4], therefore it is imperative to know whether certain variations confer any survival advantage to the virus. HIV-1 subtype-specific differentiations with respect to transmission, replication and progression of the disease have been reported previously [5,6]. Recently, we have reported an in-vitro study of Trans-Activator of Transcription (Tat) subtypes and demonstrated that TatB activates the HIV-1 subtype-C Long Terminal Repeat (LTR) promoter greater than TatC due to differential Trans-Activation Response element (TAR) interaction [7,8].

Tat is a 12 kD major regulatory transcription protein, encoded by exon-1 (1–72 amino acids (aa)) and exon-2 (73–101 aa) of the Tat gene and it contributes to several pathological symptoms of HIV-1 infection and replication [9,10,11]. Tat binds to nascent leader RNA and augments the HIV-1 promoter for transcription of viral genes [12]. Tat is made from a multiple spliced viral mRNA and expressed during early stages of viral infection [13]. The Nuclear Localization Signal (NLS) of Tat constitutes amino acid sequences of RKKRRQRRR, which enables Tat to accumulate in the nucleolus [14]. The trans-activation domain of Tat (1–48 aa) is implicated in interactions with several cellular proteins [15], namely CyclinT1, Sp1, Histone Acetyl Transferase (HAT) and Protein Kinase R (PKR), while the basic domain of Tat (49–72 aa) is involved in binding to TAR [16], nuclear localization [17] and plasma membrane permeability [18]. The second exon (73–101 aa) defines a separate domain that is involved in cell death [19]. In addition, Tat is involved in RNAi silencing suppressor (RSS) activity [20] and induction of cell death in T cells [21].

RNA silencing is a eukaryotic posttranscriptional gene regulation mechanism and innate defense to abrogate virus infections [22]. Understanding complex interplay between siRNAs, micro-RNAs, and RSS with host antiviral immunity has revolutionized our current thinking of replication of many viruses including HIV-1, influenza and other viruses, which have widespread implications for viral replication and pathogenesis [23,24]. Viruses have developed strategies to abrogate this pathway by interacting with the proteins involved, namely a double-stranded RNA-specific endoribonuclease (DICER) and Drosha [25]. In general, human cells evoke a strong small interfering RNA (siRNA) or micro RNA (miRNA) mediated response upon HIV-1 infection that could control HIV-1 replication [26]. Tat derived from HIV-1 pNL4-3-subtype B has been shown to possess RSS activity by interfering with the cellular siRNA/microRNA machinery by interacting with the enzyme DICER [27]. RNA silencing-mediated translation repression plays a strategic role in determining the viral set-point in a newly HIV-1-infected patient [20]. The differential abilities of Tat subtypes in various viral activities is due to its differences in the genetic pattern, which in turn modulate its functions [28], however, it is not known how these genetic differences would play a role in the RSS activity. The majority of HIV-1 infections are due to genetic subtype C in India underscores the importance of determining the RSS potentials of natural Tat variants with the control subtypes TatB and TatC.

HIV-1 disease is characterized by increasing viral loads [29] and elevated levels of cell death, leading to progressive loss of CD4 T-cells [30], destruction of the immune system and increased immune escape through manipulation of the cellular apoptotic machinery [31]. In-vitro studies have shown that Tat promotes cell death during HIV-1 infection via intrinsic pathway in a number of human cell lines [32]. Tat is secreted out from the infected cells [32], which are then endocytosed by neighboring cells [33] to cause cell death. The RGD motif of subtype B Tat exon-2 is involved in increased cell death [34]. The glutamine-rich region of HIV-1 Tat protein is involved in the Tat-mediated cell death of T-cells [35]. The cysteine-rich domain and basic domains of HIV-1 Tat peptides inhibit angiogenesis and induce endothelial cell death [36]. The core region has a highly conserved phenylalanine (F) and, in combination with the end of cysteine-rich region, is important in tubulin binding and cell death [37]. Numerous evidences suggest that Tat plays an important role in mediating cell death, which is associated with immune suppression [38,39,40,41], although other HIV-1 proteins (GP120, nef, vpr, vpu, and protease) have also been shown to induce cell death [42,43,44,45]. Mechanisms by which Tat has been reported to induce cell death include upregulation of several apoptotic proteins Fas ligand, Bax, caspase 8, Receptor-binding cancer antigen expressed on SiSo cells (RCAS)-1, TNF-related apoptosis-inducing ligand (TRAIL), activation of cyclin-dependent kinases, microtubule alteration and inhibition of expression of manganese-dependent superoxide dismutase [39,46,47].

HIV-1 subtype-specific differential activities including the ability to activate HIV-1 LTR promoters have been shown [1,28]. However, the subtype-specific abilities of Tat and natural Tat variants for RSS activity and cell death have not been explored. Here, we aimed to determine the cell death potential and RSS activity of natural subtype TatC variants with the control subtypes TatB and TatC.

## 2. Materials and Methods

### 2.1. Plasmids and Antibodies

HIV-1 subtype TatB (pNL4-3 GenBank accession number AF324493) and TatC (Indian isolate 93IN905 GenBank accession number AF067158) received from NIH AIDS Reagent Program and Indian Tat variants (TatN12, TatD60, TatVT6) from our previous study [7] were cloned into mammalian expression vector pCMV-Myc (Clonetech, Mountain View, CA, USA) under the cytomegalovirus (CMV) promoter for functional characterization described in this study. The RNAi-Ready-pSIREN-RetroQ-ZsGreen; referred as Retro-Q vector (the vector map and sequence has included in the Supplemental Figure S1) was obtained from Clonetech. Tat antibody (NIH AIDS Reagent Program), GAPDH antibody and Anti-IgG conjugated to Horse-Radish Peroxidase (HRP) were obtained from Cell Signaling Technology (Danvers, MA, USA). Anti-cytochrome C antibody was obtained from Biolegend (San Diego, CA, USA).

### 2.2. Cell Culture and Transfection

HEK293T cells and TZM-bl cells (also known as JC53BL-13) (NIH AIDS Reagent Program) were maintained in Dulbecco’s modified Eagle’s medium (DMEM) (Himedia Laboratories, Mumbai, India) supplemented with l-glutamine and sodium pyruvate, fetal bovine serum (FBS) (10%), penicillin (100 U/mL) and streptomycin (0.1 mg/mL) at 37 °C in the presence of 5% CO_2_. TZM-bl is a genetically engineered HeLa cell line that expresses CD4, CXCR4 and CCR5 and contains Tat-inducible Luc and b-Gal reporter genes. The Jurkat cells were maintained in RPMI supplemented with l-glutamine, 10 mM HEPES, 23.8 mM sodium carbonate, penicillin (100 U/mL) and streptomycin (0.1 mg/mL) and 10% FBS. All cell types were transfected with lipofectamine 2000 (Invitrogen, Carlsbad, CA, USA) in serum free DMEM media. For each transfection, 1 μg of vector DNA was diluted in 250 μL serum free media; 1:3 ratio of lipofectamine with vector DNA was used. Cells were processed for various assays at 24 h post-transfection. The empty control vector DNA was used to normalize equal amounts of vector DNA (Tat cloned into) in each transfection.

### 2.3. Immuno-Blotting

HEK293T cells (1 μg plasmid DNA/well in 1 × 10^6^ cells) were transfected with Tat variants (TatN12 or TatVT6 or TatD60) or Tat subtypes (TatB or TatC) or empty pCMV-Myc vector. Jurkat cells (1 μg plasmid DNA/well in 1 × 10^6^ cells) were transfected with Tat subtypes TatB or TatC or empty pCMV-Myc vector as a control. After 24 h post-transfection, cells were harvested, and total protein was extracted using RIPA lysis buffer (Invitrogen), supplemented with protease inhibitor (Invitrogen) and phosphatase inhibitor tablets (Invitrogen). Protein concentrations in supernatants were measured by the bicinchoninic acid (BCA) assay (Thermo Fisher Scientific, Waltham, MA, USA), Equal amounts of protein were loaded and separated in 8–10% SDS-acrylamide resolving gels and transferred to a nitrocellulose membrane (BIORAD, Hercules, CA, USA) using standard methods. The membranes were incubated with Tat antibody (1:2000 dilution), anti-cytochrome C antibody (1:2500 dilution) and GAPDH (1:5000 dilution) overnight at 4 °C after one hour of blocking with nonfat milk of 5%, and then washed three times with PBS with 0.1% Tween 20. The membranes were finally incubated with an HRP-linked secondary IgG antibody (1:5000 dilution). The bound antibody was detected with the ECL immunoblot system (Pierce). The GAPDH was used as a loading control and the expression of proteins was normalized with the amount of GAPDH. The empty pCMV-Myc vector was used as a control in all the experiments, and the experiment was repeated three times for confirmation of the result.

### 2.4. Reverse Transcriptase-PCR

HEK293T cells (1 μg plasmid DNA/well in 1 × 10^6^ cells) were transfected with Tat variants (TatN12 or TatVT6 or TatD60) or Tat subtypes (TatB or TatC) or empty pCMV-Myc vector. After 24 h of transfection, RNA was isolated from the transfected cell lysates using Trizol (Sigma, St. Louis, MO, USA) and then reverse transcribed using a RT-PCR kit (Promega, Madison, WI, USA). One μg of template RNA and 1 μM terminal primers were combined in a 5 μL reaction volume. The mix was thermally denatured at 70 °C for 5 mins and subsequently chilled on ice for 3 mins. The reverse transcription reaction mixture of volume 15 μL was set with nuclease-free water, 1× reaction buffer, 1 μL RT enzyme, 6 mM magnesium chloride, 0.5 mM dNTPs and 1 U ribonuclease inhibitor RNasin. The following steps were carried out on PCR machine, an initial annealing at 25 °C for 5 mins, incubation at 42 °C for 1 hour and at 75 °C for 15 mins. The cDNA was used for amplification of Tat using Tat-specific primers as follows:

FP: 5′-ATGGAGCCAGTAGATCCTAACCTA-3′

RP: 5′-TTGCTTTGATATAAGATTTTGATGATCCT-3′

Beta-actin was used as a loading control. All experiments were repeated three times for confirmation of the result.

### 2.5. RNAi Silencing Suppressor Assay

To study the RSS activities of Tat subtypes and our Tat variants, we utilized a sensitive HIV-1 replicon based mammalian cell assay that was developed in our laboratory to establish the RSS activity of Sars7 protein [48]. In this study, we used a similar mammalian system vector, a bi-cistronic gene vector Retro-Q that encodes the GFP under CMV promoter, and shRNA under the U6 promoter, as described detail [48]. In this vector, shRNA for luciferase (an unrelated shRNA control referred as shRNA-luc) was used as a control, while shRNA for green fluorescence (nucleotide [nt] positions 973 to 994 referred as shRNA-GFP) was used to abrogate GFP expression. The negative control was un-transfected cells, the positive control (showing 80% GFP expression) was transfected cells with shRNA-luc and the RSS control (showing 50% silencing of GFP expression) was transfected cells with shRNA-GFP or shRNA-GFP+empty pCMV-Myc. The pCMV-Myc vector used for cloning all Tat variants and subtypes. The shRNA-GFP (0.5 μg in 1 mL) was co-transfected either with Tat variants (TatN12 or TatVT6 or TatD60) or Tat subtypes (TatB or TatC) or empty pCMV-Myc vector in HEK293T cells (1 μg plasmid DNA/well in 1 × 10^6^ cells). After 24 h of co-transfection, cell lysates were analyzed for GFP expression using flow cytometry (BD-LSRII) and confocal microscopy (Olympus FluoView FV1000). The empty pCMV-Myc vector was used as a control to determine the differential levels of RSS activity in each subtypes and variants. This experiment was repeated three times for confirmation of the results.

### 2.6. Propidium Iodide (PI) Staining

HEK293T or TZM-bl cells (1 μg plasmid DNA/well in 1 × 10^6^ cells) were transfected with Tat variants (TatN12 or TatVT6 or TatD60) or Tat subtypes (TatB or TatC) or empty pCMV-Myc vector. For cell death analysis, infected cells were collected and washed in 1× PBS. Finally, the cells were re-suspended in 1× PBS containing PI at a final concentration of 10 µg/mL. The cells were analyzed on flow-cytometer (BD-LSRII) for PI incorporation to measure cell death. The cell death rate was analyzed using FlowJo software. PI-stained cells fall in the PE channel; therefore, these cells were counted in PE logH versus SSC linH. The unstained cells were used to make gating or quadrant with PI stained cells. The number of cells stained with PI were counted and expressed in percentage. The level of cell death was measured by comparing the empty pCMV-Myc vector with subtype TatB, C and Tat variants. The experiment was repeated three times for confirmation of the results.

### 2.7. Statistical Analysis

All the data were analyzed using GraphPad Prism Version 8.0.1 (LaJolla, CA, USA). For all the measurements, the student *t*-test was used to assess the statistical significance between groups. A statistically significant difference was considered at the level of *p* < 0.05, *** highly significant (*p* < 0.0005), ** moderately significant (*p* < 0.005), * significant (*p* < 0.05).

### 2.8. Data Availability

Authors declare that the data supporting the findings of this study are available within the paper and its Supplementary Information Files. The data are also available from the corresponding author upon request. The nucleotide sequences are available at GenBank with the accession numbers: HQ110625, FJ432073, HQ110614.

## 3. Results

### 3.1. Expression of Tat Subtypes and Variants at Protein and RNA Levels

**Figure 1:**
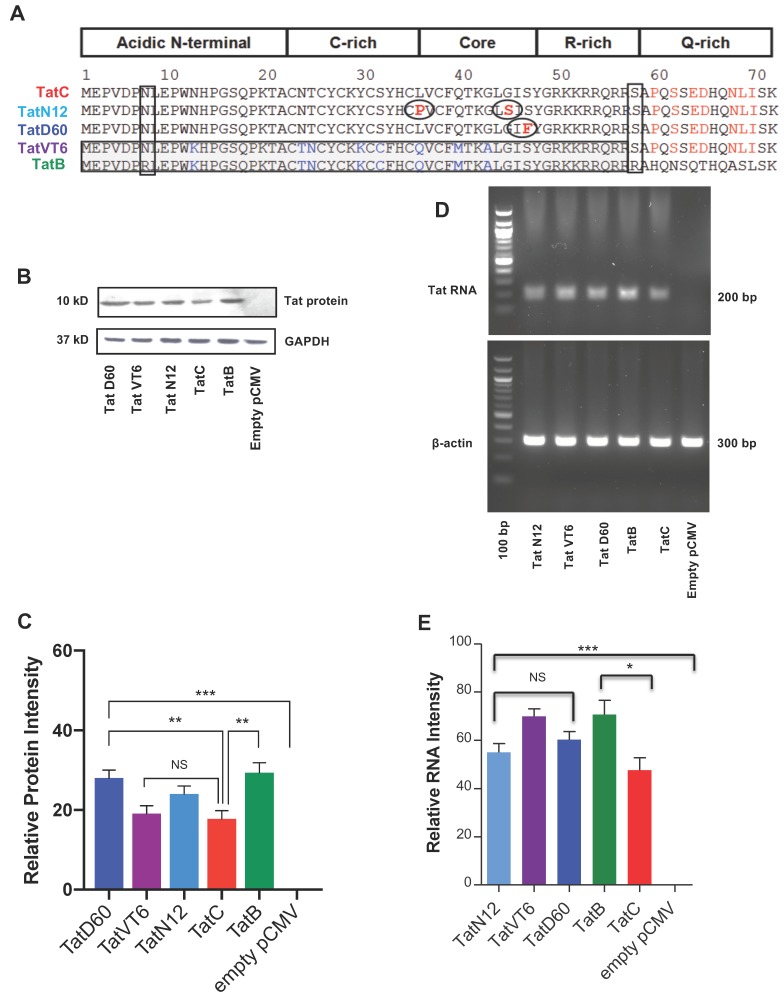
Expression of Tat subtypes and variants at protein and transcriptional level. Panel (**A**) A comparison of the sequence between TatB subtype (A HIV-1 NL4-3 Infectious Molecular Clone (pNL4-3); accession No. U26942.1) with the Indian isolate TatC (clone 93IN905; accession No.AF067158) revealed conserved (9 aa) change in the amino acid sequences. Our TatC variants, TatN12 (accession No. HQ110625), a subtype C variant with Leucine35Proline and Glycine44Serine; TatD60 (accession No. HQ110614), also a subtype C variant with Glutamic_acid9Lysine, Serine46Phenylalanine and Serine61Arginine; and TatVT6 (accession No. FJ432073), a B/C recombinant having N-terminal, C-rich, Core and R-rich regions from subtype TatB whereas the Q-rich region was from subtype TatC. Panel (**B** and **C**) Tat variants (TatN12 or TatVT6 or TatD60) or Tat subtypes (TatB or TatC) or empty pCMV-Myc vector were checked for intracellular expression by transfecting (1 μg plasmid DNA/well in 1 × 10^6^ cells) on Human embryonic kidney 293 expresses a mutant version of the SV40 large T antigen (HEK293T) cells and were measured by western blot using Tat antibody. Panel (**D** and **E**) Upon transfection with Tat variants (TatN12 or TatVT6 or TatD60) or Tat subtypes (TatB or TatC) or empty pCMV-Myc vector on HEK293T cells (1 μg plasmid DNA/well in 1 × 10^6^ cells), the RNA expression was monitored by Reverse Transcriptase PCR (RT-PCR). The relative protein and RNA intensity of Tat variants was calculated by normalizing with the amount of beta-actin expressed in the corresponding Tat variants after deducing the expression of empty pCMV-Myc vector. The level of GAPDH was used as a loading control and the empty pCMV-Myc vector was used as a negative control. Multiple-group comparisons were performed using one-way analysis of variance (ANOVA) followed by student *t*-test and are presented as mean ± S.E.M. *** Highly significant (*p* < 0.0005), ** moderately significant (*p* < 0.005), * significant (*p* < 0.05), NS is non-significant. All experiments were repeated three times.

Five Tat proteins were analyzed based on their genetic similarity to represent subtype B, subtype C, or variations of subtype B and C. TatB, the representative Tat from subtype B; TatC, the representative Tat from subtype C; TatN12, a subtype C variant; TatD60, also a subtype C variant; and TatVT6, a B/C recombinant were used in this study. These variants showed more than 80% sequence similarity to subtype TatC. The unique mutations in these variants as compared to TatC are shown in the Figure 1A. The amino acid sequence comparisons between subtype TatB (pNL4-3) and TatC (93IN905) revealed 9 amino acid changes in almost all domains of Tat exon 1 sequence (Figure 1A). Tat protein expression was measured after 24 h of transfection on human embryonic kidney (HEK293T) cells with Tat variants and Tat subtypes by western blotting. The empty pCMV-Myc vector was used as a control to measure the relative protein intensity. TatB protein was expressed higher (*p* < 0.005) than that of TatC. TatN12 and TatVT6 proteins were expressed similarly to TatC. TatD60 was expressed at a higher level than other TatC variants, namely TatN12 and TatVT6, and also higher (*p* < 0.005) than TatC (Figure 1B,C). All Tat variants and subtypes were well expressed (*p* < 0.0005) at the translational level which were normalized to the expression levels of control GAPDH and the relative protein intensity was calculated from the control pCMV-Myc vector. Tat RNA expression was measured after 24 h of transfection with Tat variants on HEK293T cells by Reverse Transcriptase-PCR (RT-PCR). The empty pCMV-Myc vector was used as a control to measure the relative RNA intensity. All Tat variants and subtypes were well expressed (*p* < 0.0005) at the transcriptional level which were normalized to the expression levels of control beta-actin and the relative RNA intensity was calculated from the control plasmid Cytomegalovirus expressing an N-terminally Myc-tagged protein (pCMV-Myc) vector. We observed a less significant difference between TatB and TatC subtypes (*p* < 0.05), however there were no significant changes between TatC and Tat variants indicating that the genetic variations in Tat variants might not be affected at the RNA expressional levels (Figure 1D,E). These observed differences at the protein and RNA level with respect to subtypes and variants might be due to the amino acid variations found in Tat proteins.

### 3.2. RNAi Silencing Suppression (RSS) Activity of Tat Subtypes and Variants

**Figure 2:**
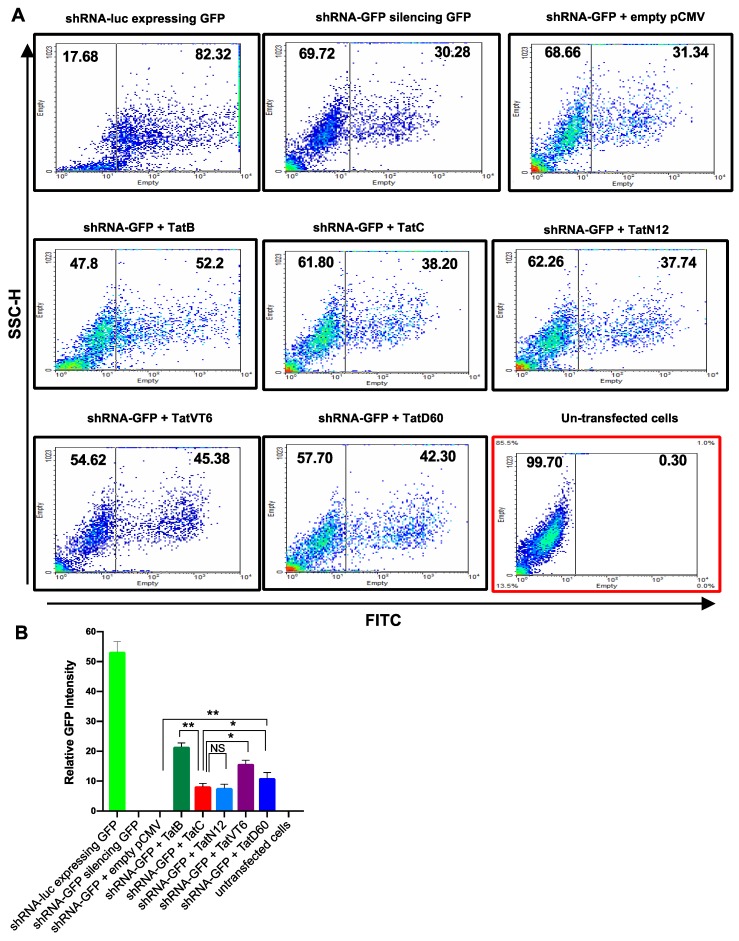
A short hairpin RNA (shRNA) suppressor activity of Tat variants and subtypes by flow-cytometry. Panel (**A** and **B**) HEK293T cells (1 μg plasmid DNA/well in 1 × 10^6^ cells) were co-transfected with shRNA-GFP (0.5 μg in 1 mL) and Tat variants (TatN12 or TatVT6 or TatD60) or Tat subtypes (TatB or TatC) or empty pCMV-Myc vector. The 1:1 ratio of shRNA-GFP and Tat constructs was used for transfection. After 24 h of transfection, cells were counted for GFP-expressing cells by flow-cytometry (BD-LSRII) using the Green Fluorescent Protein (GFP) under Fluorescein isothiocyanate (FITC) channel versus a Side Scatter-Height (SSC-H). The flow data was analyzed using FlowJo 10.3 software. In the plot, the x-axis represents FITC (GFP) and the y-axis represents SSC-H. The relative GFP intensity of Tat variants was calculated by normalizing with the expression of empty pCMV-Myc vector. The un-transfected cells were used as a control to make gating for the transfected cells. The cells treated with shRNA-GFP vector + empty pCMV-Myc vector was used as a control to show that there was no effect of pCMV-Myc vector in RSS activity. Multiple-group comparisons were performed using one-way analysis of variance (ANOVA) followed by student *t*-test and are presented as mean ± S.E.M.), ** moderately significant (*p* < 0.005), * significant (*p* < 0.05), NS is non-significant. All experiments were repeated three times.

To understand the effect of Tat on RNAi, we co-transfected HEK293T cells with the plasmid Retro-Q, which co-expresses GFP and a chosen short hairpin RNA (shRNA), while a pCMV plasmid which expresses the chosen Tat protein. Transfection with the positive control (shRNA-luc) in HEK293T cells resulted in almost 80% cells showing green fluorescence, while the negative control (un-transfected cells) showed no fluorescence. The RSS control was transfected with shRNA-GFP or shRNA-GFP+empty pCMV-Myc, showed 50% reduction in green fluorescence. When shRNA-GFP was co-transfected with either TatB, or TatC, or Tat variants, the number of GFP-expressing cells was increased. TatB exhibited a significantly (*p* < 0.005) higher level of reversal of shRNA inhibition (40%) compared to TatC, which only showed 13% RSS activity (Figure 2A,B). These results indicate the role of Tat as a RSS activator in our system. TatC variant, TatN12 showed 11% RSS activity which was similar to TatC. TatVT6 and TatD60 showed 27% and 21% respectively, which was found to be statistically higher (*p* < 0.05) than TatC (Figure 2A,B) and the data was normalized with the protein expression of corresponding Tat subtypes and variants indicating varying levels of RSS activity (Supplemental Figure S5A). All Tat variants and subtypes were found to show varying levels (*p* < 0.005) of RSS activity depending on the types of Tat. The relative GFP intensity was calculated from the expression level of RSS control (shRNA-GFP + empty pCMV-Myc).

**Figure 3:**
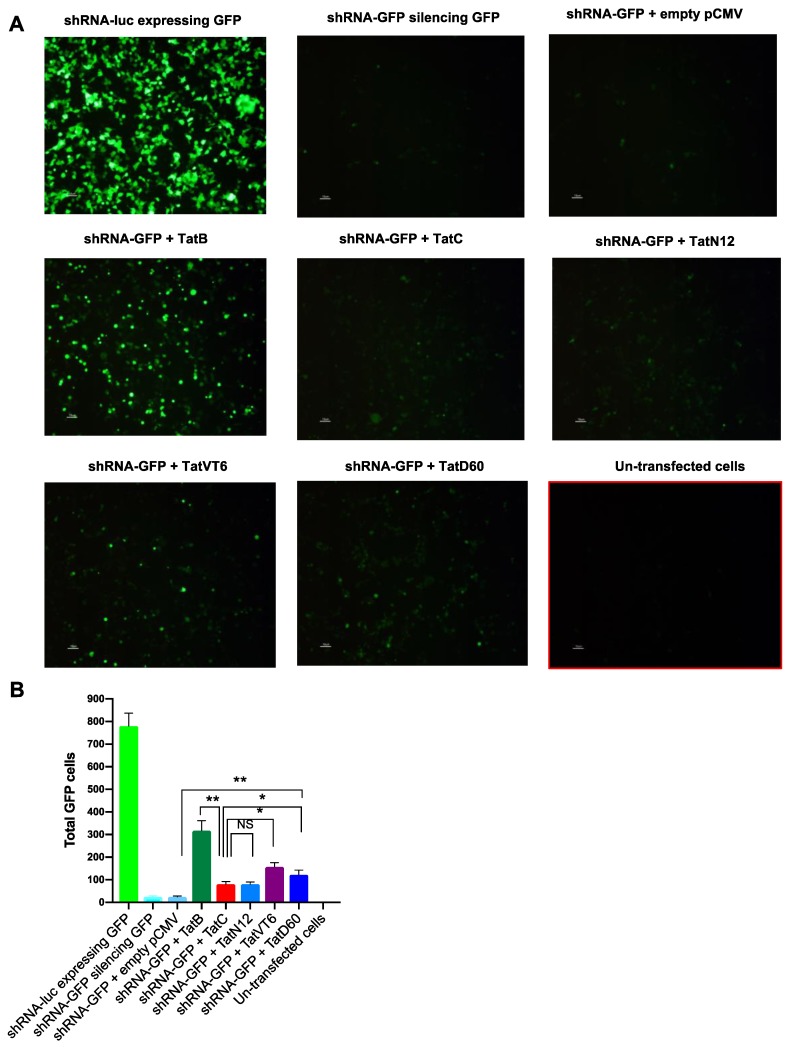
shRNA suppressor activity of Tat variants and subtypes by microscopy. Panel (**A** and **B**) HEK293T cells (1 μg plasmid DNA/well in 1 × 10^6^ cells) were co-transfected with shRNA-GFP (0.5 μg in 1 mL) and Tat variants (TatN12 or TatVT6 or TatD60) or Tat subtypes (TatB or TatC) or empty pCMV-Myc vector. The 1:1 ratio of shRNA-GFP and Tat constructs was used for transfection. After 24 h of transfection, cells were imaged in a confocal microscope (Olympus FluoView FV1000) for GFP-expressing cells under the magnification of 10×. The GFP-expressing cells were counted using ImageJ software (in Cell Counter plugin). The total number of GFP cells were plotted against each transfected condition. The un-transfected cells were used as a control for the transfected cells. The cells treated with shRNA-GFPvector + empty pCMV-Myc vector was used as a control to show that there was no effect of our expression pCMV-Myc vector in RSS activity. More than 10 to 15 regions were captured for each transfected condition and the accumulative data were plotted. Multiple-group comparisons were performed using one-way analysis of variance (ANOVA) followed by student *t*-test and are presented as mean ± S.E.M.), ** moderately significant (*p* < 0.005), * significant (*p* < 0.05), NS is non-significant. All experiments were repeated three times.

We further validated our data with confocal microscopy image analysis. TatB significantly (*p* < 0.005) restored GFP cells (45%) as compared to TatC which restored 11% of GFP cells. TatN12, TatD60 and TatVT6 also restored 10% to 20% GFP cells. Among Tat variants, TatVT6 showed moderately higher level (*p* < 0.005) of GFP cells (20%) than TatC while TatD60 restored 15% GFP cells which was slightly higher (*p* < 0.05) than TatC (Figure 3A,B) and the data was normalized with the protein expression of corresponding Tat subtypes and variants indicating varying levels of RSS activity (Supplemental Figure S5B). The reduction in shRNA level was associated with a corresponding increase in restoration percentage of GFP cells by Tat variants and subtypes. The empty pCMV-Myc was co-transfected with shRNA-GFP that showed similar values to the cells transfected with shRNA-GFP indicating that percentage of reversal of shRNA inhibition was not affected by the pCMV-Myc vector. The GFP control was included in the experiment (Supplemental Figure S4) to show that there was no effect of Tat on GFP-driven expression and to have similar levels of transfection efficiencies of all transfected cells.

### 3.3. Induction of Cell Death by Tat Subtypes and Variants

**Figure 4:**
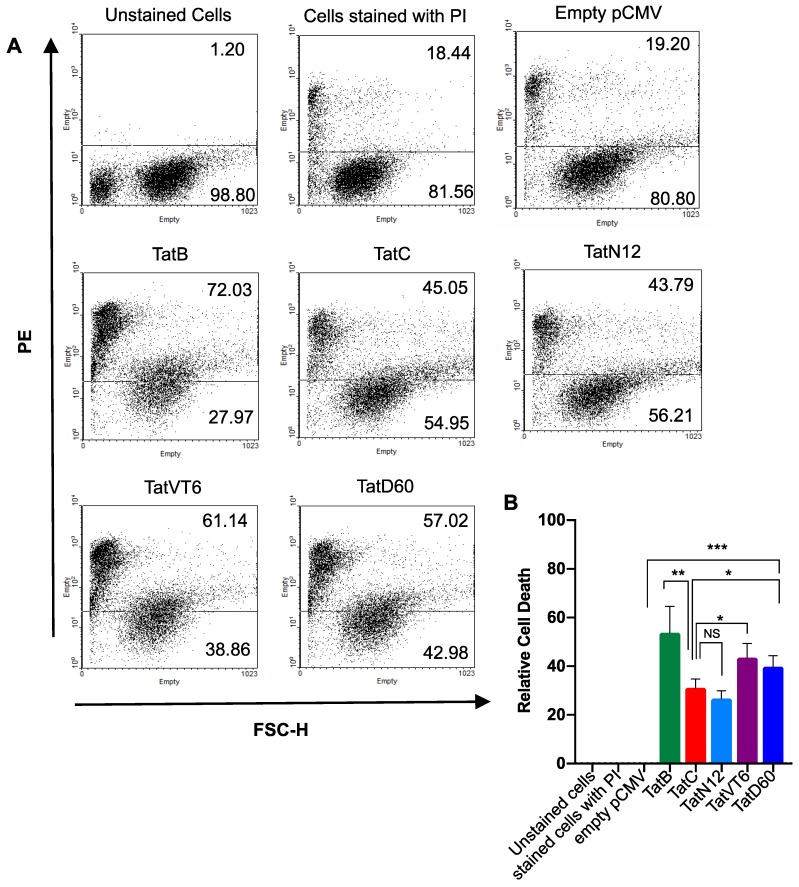
Tat subtypes and variants induced cell death in HEK293T cells. Panel (**A** and **B**) HEK293T cells (1 μg plasmid DNA/well in 1 × 10^6^ cells) were transfected with Tat variants (TatN12 or TatVT6 or TatD60) or Tat subtypes (TatB or TatC) or empty pCMV-Myc vector. After 24 h of transfection, cells were stained with PI (10 μg/mL) for 20 to 30 mins on Ice under dark condition. The flow cytometry (BD-LSRII) was performed to determine cell death and the data was analyzed using FlowJo 10.3 software. PI stained cells fall in the PE channel. In the plot, the X-axis represents FSC-H and the Y-axis represents PE. The un-stained cells were used as a control to make gating for the transfected cells. The cells treated with empty pCMV-Myc vector were used as a control to show that there was no effect of our expression pCMV-Myc vector in causing cell death. The relative cell death of Tat variants was measured by subtracting empty pCMV-Myc vector values. Multiple-group comparisons were performed using one-way analysis of variance (ANOVA) followed by student *t*-test and are presented as mean ± S.E.M. *** Highly significant (*p* < 0.0005), ** moderately significant (*p* < 0.005), * significant (*p* < 0.05), NS is non-significant. All experiments were repeated three times.

To investigate the ability of Tat to induce cell death, in-vitro cell death analysis was carried out using HEK293T cells transfected with Tat variants, and propidium iodide (PI) release was measured. The induced cell death by Tat was measured 24 h after transfection by PI staining followed by flow-cytometry with the empty pCMV-Myc vector used as a control. TatB showed significantly (*p* < 0.005) higher level of cell death of 53% than TatC which showed comparatively less cell death of 28%. The level of cell death induced by TatN12 was 24%, which was comparably similar to TatC. TatD60 showed relatively higher (*p* < 0.05) level of cell death (38%) than TatC. TatVT6 induced cell death by 42%, which was higher (*p* < 0.005) than TatC (Figure 4A,B) and the data was normalized with the protein expression of corresponding Tat subtypes and variants indicating differential cell death (Supplemental Figure S5C). All Tat variants and subtypes were found to show varying levels (*p* < 0.0005) of cell death depending on the types of Tat.

Similarly, cell death induced by Tat variants and subtypes was measured on TZM-bl cells by PI staining followed by flow-cytometry with the empty pCMV-Myc vector was used as a control. Once again, it was confirmed that TatB exhibited higher (*p* < 0.005) level of 50% than TatC which resulted in 25% of cell death. TatN12 showed 23% of cell death which was relatively similar level to TatC. TatD60 and TatVT6 exhibited 32% and 36% of cell death respectively which was higher (*p* < 0.005) than TatC (Supplemental Figure S2A,B) and the data was normalized with the protein expression of corresponding Tat subtypes and variants indicating differential cell death (Supplemental Figure S5D). The cells treated with empty pCMV-Myc showed similar levels of cell death to that of un-transfected cells (PI stained) indicating that the pCMV-Myc vector didn’t affect the cell death.

Additionally, to understand whether Tat subtypes use the same mitochondria-dependent pathway to cause cell death, we measured levels of cell death induced by two different genetic subtypes that could be co-related with the release of cytochrome C [35]. Under identical conditions, we observed a 2–3-fold increase in release of cytochrome-C with subtype-B Tat as compared to that of subtype-C Tat (Supplemental Figure S3). As expected, the untreated control Jurkat T-cells showed no cytoplasmic release of cytochrome–C.

## 4. Discussion

Viruses have evolved various strategies to survive and develop infection inside the host cells by modifying host processes, such as cell death induction and inhibition, or by abrogating anti-viral factors, such as APOBEC, and RNA interference (RNAi) [49,50]. HIV-1 Tat is a poly-functional protein that governs a wide range of cellular and viral gene functions [34,51]. Tat is reported to act as suppressor of RNAi by binding to DICER (RNA-specific endoribonucleases), thereby causing RNAi suppression [27,52]. It has been demonstrated that Tat variants have been found to show widely varying potential for their ability to activate HIV-1 LTR promoter [1,27,53,54] via TAR interactions [7] through hydrogen bonds [8]. However, there is no data available to address whether these Tat variants and subtypes differ in their ability as a suppressor of RNAi or mediated cell death. This study aimed to find the role of natural Tat variants and Tat subtypes such as TatB and TatC in RNAi-suppressor activity and cell death induction to know whether the genetic determinants that impact on HIV-1 thereby potentially serve as important targets for therapeutics.

TatN12, a subtype C variant with Leu35Pro and Gly44Ser; TatD60, also a subtype C variant with Glu9Lys, Ser46Phe and Ser61Arg; and TatVT6, a B/C recombinant have been characterized before for HIV-1 transactivation and other Tat functions [7]. Tat variants were chosen based on the similarities in their genetic and functional activities. Before further investigation of control subtypes (TatB, TatC), our variants (TatN12, TatD60, and TatVT6) on RSS activity and cell death, we verified the expression levels of Tat at protein and RNA levels by western blotting and RT-PCR, respectively. We found TatB was better expressed at the protein level than TatC, which could be due to unique variation between these subtypes. TatVT6 was better expressed than TatC, perhaps due to the chimeric nature of TatVT6. TatB and the Tat variants showed similar levels of RNA expression, but TatC showed a slightly reduced level of RNA expression. The subtype specific variations in Tat gene would be responsible for the differences in protein production and RNA expression.

In this study, TatB showed more potent RSS activity than TatC and other Tat variants. This differential RSS activity might be due to the differential genetic signature pattern of subtypes; however, the protein expressions might also modulate the differential activities. We examined cell death by analyzing the DNA content of propidium-iodide treated cells using flow analysis and by cytochrome C release. Under identical conditions, TatC from an Indian isolate showed low levels of cell death when compared with those from subtype TatB. It is likely that reduced cell death mediated by HIV-1 TatC may result in a greater number of susceptible host cells for the virus in comparison to HIV-1 TatB infection. On other hand, Tat triggers high levels of cell death which may be critical for the virus at the end of the cell-cycle process to release virions from the human cells [21,55]. TatB showed a higher level of cell death than TatC, which could be due to variation in the cysteine-rich and glutamine-rich regions that are known to mediate cell death. TatVT6, a B/C recombinant, showed higher cell death than TatC, but lower than TatB, which could be due to a cysteine-rich region from originating from TatB and the glutamine region from TatC. This study elucidates the importance of unique polymorphisms in Tat variants (TatN12 and TatD60), as well as subtype-specific variations in cysteine and glutamine regions of TatVT6 in causing varying levels of cell death and RSS activities.

In summary, Tat subtype-specific activities displayed by HIV-1 within infected individuals might generate selective advantages with respect to RSS activity and induction of cell death. Since RSS activity and cell death are critical for viral fitness and pathogenesis, augmentation in these activities could greatly enhance HIV fitness. Subtype-specific differential activities mediated by Tat indicate that cell death may be differentially regulated by Tat subtypes to avoid premature death of an infected cell or to destroy the human cells after the release of virions. This study demonstrates how understanding subtype-specific Tat mutation will be necessary to elucidate HIV activities.

## Supplementary Materials

The following are available online at https://www.mdpi.com/1999-4915/11/11/976/s1, Figure S1: pSIREN-RetroQ-ZsGreen1 vector. Figure S2: Tat subtypes and variants induced cell death in TZM-bl cells. Figure S3: Tat subtypes induce cell death via release of cytochrome-c. Figure S4: the effect of Tat on GFP-driven expression. Figure S5: The cell death and RSS activity normalized with the protein expression of corresponding Tat subtypes and variants.
