# Supplementary material for: In-Vitro Subtype-Specific Modulation of HIV-1 Trans-Activator of Transcription (Tat) on RNAi Silencing Suppressor Activity and Cell Death
Source: Viruses. 2019 Oct 23;11(11):976. doi: 10.3390/v11110976 (PMC6893708; doi:10.3390/v11110976)
Supplement: Supplementary file 1 [file viruses-11-00976-s001.zip › S1.pdf]

Sequence: pSIREN-RetroQ-ZsGreen1 (1).dna (Linear / 6562 bp)
Enzymes: Unique 6+ Cutters (33 of 653 total)
Features: 15 total
Primers: 1 total

Unique Cutters **Bold**

Linearized retroviral vector for inserting an shRNA cassette that will be co-expressed with ZsGreen1.

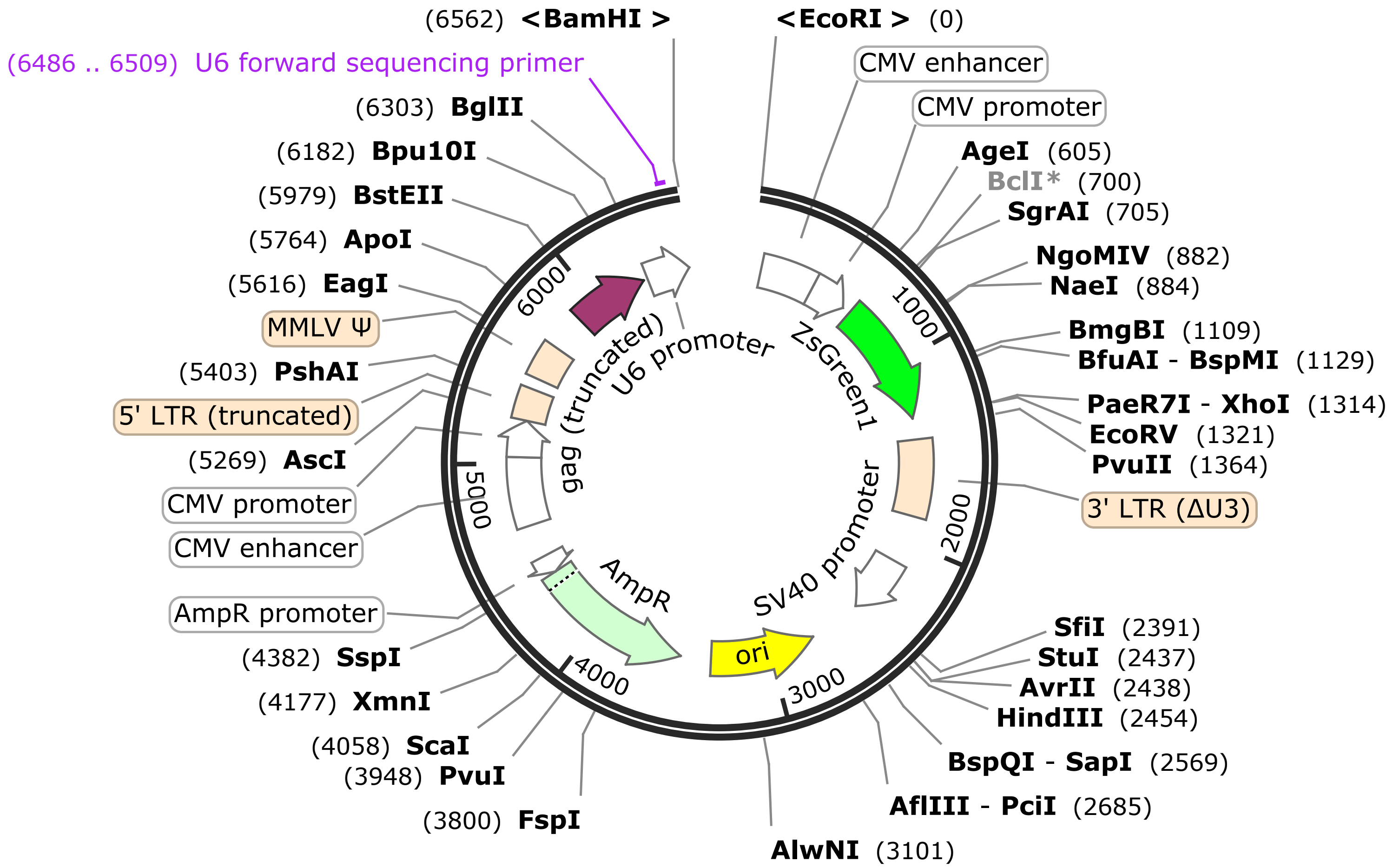

pSIREN-RetroQ-ZsGreen1
6562 bp

```
AATTCTAGTTATTAATAGTAATCAATTACGGGGTCATTAGTTCAT      45
AGCCCATATATGGAGTTCCGCGTTACATAACTTACGGTAAATGGC      90
CCGCCTGGCTGACCGCCCAACGACCCCCGCCCATTGACGTCAATA     135
ATGACGTATGTTCCCATAGTAACGCCAATAGGGACTTTCCATTGA     180
CGTCAATGGGTGGAGTATTTACGGTAAACTGCCCACTTGGCAGTA     225
CATCAAGTGTATCATATGCCAAGTACGCCCCCTATTGACGTCAAT     270
GACGGTAAATGGCCCGCCTGGCATTATGCCCAGTACATGACCTTA     315
TGGGACTTTCCTACTTGGCAGTACATCTACGTATTAGTCATCGCT     360
ATTACCATGGTGATGCGGTTTTGGCAGTACATCAATGGGCGTGGA     405
TAGCGGTTTGACTCACGGGGATTTCCAAGTCTCCACCCCATTGAC     450
GTCAATGGGAGTTTGTTTTGGCACCAAAATCAACGGGACTTTCCA     495
AAATGTCGTAACAACTCCGCCCCATTGACGCAAATGGGCGGTAGG     540
CGTGTACGGTGGGAGGTCTATATAAGCAGAGCTGGTTTAGTGAAC     585
CGTCAGATCCGCTAGCGCTACCGGTCGCCACCATGGCCCAGTCCA     630
AGCACGGCCTGACCAAGGAGATGACCATGAAGTACCGCATGGAGG     675
GCTGCGTGGACGGCCACAAGTTCGTGATCACCGGCGAGGGCATCG     720
GCTACCCCTTCAAGGGCAAGCAGGCCATCAACCTGTGCGTGGTGG     765
AGGGCGGCCCCTTGCCCTTCGCCGAGGACATCTTGTCCGCCGCCT     810
TCATGTACGGCAACCGCGTGTTCACCGAGTACCCCCAGGACATCG     855
TCGACTACTTCAAGAACTCCTGCCCCGCCGGCTACACCTGGGACC     900
GCTCCTTCCTGTTCGAGGACGGCGCCGTGTGCATCTGCAACGCCG     945
ACATCACCGTGAGCGTGGAGGAGAACTGCATGTACCACGAGTCCA     990
AGTTCTACGGCGTGAACTTCCCCGCCGACGGCCCCGTGATGAAGA    1035
AGATGACCGACAACTGGGAGCCCTCCTGCGAGAAGATCATCCCCG    1080
TGCCCAAGCAGGGCATCTTGAAGGGCGACGTGAGCATGTACCTGC    1125
TGCTGAAGGACGGTGGCCGCTTGCGCTGCCAGTTCGACACCGTGT    1170
ACAAGGCCAAGTCCGTGCCCCGCAAGATGCCCGACTGGCACTTCA    1215
TCCAGCACAAGCTGACCCGCGAGGACCGCAGCGACGCCAAGAACC    1260
AGAAGTGGCACCTGACCGAGCACGCCATCGCCTCCGGCTCCGCCT    1305
TGCCCTAACTCGAGATATCAGTGGTCCAGGCTCTAGTTTTGACTC    1350
AACAATATCACCAGCTGAAGCCTATAGAGTACGAGCCATAGATAA    1395
AATAAAAGATTTTATTTAGTCTCCAGAAAAAGGGGGGAATGAAAG    1440
ACCCCACCTGTAGGTTTGGCAAGCTAGCTTAAGTAACGCCATTTT    1485
GCAAGGCATGGAAAAATACATAACTGAGAATAGAGAAGTTCAGAT    1530
CAAGGTCAGGAACAGATGGAACAGGGTCGACCCTAGAGAACCATC    1575
AGATGTTTCCAGGGTGCCCCAAGGACCTGAAATGACCCTGTGCCT    1620
TATTTGAACTAACCAATCAGTTCGCTTCTCGCTTCTGTTCGCGCG    1665
CTTCTGCTCCCCGAGCTCAATAAAAGAGCCCACAACCCCTCACTC    1710
GGGGCGCCAGTCCTCCGATTGACTGAGTCGCCCGGGTACCCGTGT    1755
ATCCAATAAACCCTCTTGCAGTTGCATCCGACTTGTGGTCTCGCT    1800
GTTCCTTGGGAGGGTCTCCTCTGAGTGATTGACTACCCGTCAGCG    1845
GGGGTCTTTCATTTGGGGGCTCGTCCGGGATCGGGAGACCCCTGC    1890
CCAGGGACCACCGACCCACCACCGGGAGGTAAGCTGGCTGCCTCG    1935
CGCGTTTCGGTGATGACGGTGAAAACCTCTGACACATGCAGCTCC    1980
CGGAGACGGTCACAGCTTGTCTGTAAGCGGATGCCGGGAGCAGAC    2025
AAGCCCGTCAGGGCGCGTCAGCGGGTGTTGGCGGGTGTCGGGGCG    2070
CAGCCATGACCCAGTCACGTAGCGATAGCGGAGTGTAGATCCGGC    2115
TGTGGAATGTGTGTCAGTTAGGGTGTGGAAAGTCCCCAGGCTCCC    2160
```

```
CAGCAGGCAGAAGTATGCAAAGCATGCATCTCAATTAGTCAGCAA 2205
CCAGGTGTGGAAAGTCCCCAGGCTCCCCAGCAGGCAGAAGTATGC 2250
AAAGCATGCATCTCAATTAGTCAGCAACCATAGTCCCGCCCCTAA 2295
CTCCGCCCATCCCGCCCCTAACTCCGCCCAGTTCCGCCCATTCTC 2340
CGCCCCATGGCTGACTAATTTTTTTTATTTATGCAGAGGCCGAGG 2385
CCGCCTCGGCCTCTGAGCTATTCCAGAAGTAGTGAGGAGGCTTTT 2430
TTGGAGGCCTAGGCTTTTGCAAAAAGCTTACTGGCTTAACTATGC 2475
GGCATCAGAGCAGATTGTACTGAGAGTGCACCATATGCGGTGTGA 2520
AATACCGCACAGATGCGTAAGGAGAAAATACCGCATCAGGCGCTC 2565
TTCCGCTTCCTCGCTCACTGACTCGCTGCGCTCGGTCGTTCGGCT 2610
GCGGCGAGCGGTATCAGCTCACTCAAAGGCGGTAATACGGTTATC 2655
CACAGAATCAGGGGATAACGCAGGAAAGAACATGTGAGCAAAAGG 2700
CCAGCAAAAGGCCAGGAACCGTAAAAAGGCCGCGTTGCTGGCGTT 2745
TTTCCATAGGCTCCGCCCCCCTGACGAGCATCACAAAAATCGACG 2790
CTCAAGTCAGAGGTGGCGAAACCCGACAGGACTATAAAGATACCA 2835
GGCGTTTCCCCCTGGAAGCTCCCTCGTGCGCTCTCCTGTTCCGAC 2880
CCTGCCGCTTACCGGATACCTGTCCGCCTTTCTCCCTTCGGGAAG 2925
CGTGGCGCTTTCTCATAGCTCACGCTGTAGGTATCTCAGTTCGGT 2970
GTAGGTCGTTCGCTCCAAGCTGGGCTGTGTGCACGAACCCCCCGT 3015
TCAGCCCGACCGCTGCGCCTTATCCGGTAACTATCGTCTTGAGTC 3060
CAACCCGGTAAGACACGACTTATCGCCACTGGCAGCAGCCACTGG 3105
TAACAGGATTAGCAGAGCGAGGTATGTAGGCGGTGCTACAGAGTT 3150
CTTGAAGTGGTGGCCTAACTACGGCTACACTAGAAGGACAGTATT 3195
TGGTATCTGCGCTCTGCTGAAGCCAGTTACCTTCGGAAAAAGAGT 3240
TGGTAGCTCTTGATCCGGCAAACAAACCACCGCTGGTAGCGGTGG 3285
TTTTTTTGTTTGCAAGCAGCAGATTACGCGCAGAAAAAAAGGATC 3330
TCAAGAAGATCCTTTGATCTTTTCTACGGGGTCTGACGCTCAGTG 3375
GAACGAAAACTCACGTTAAGGGATTTTGGTCATGAGATTATCAAA 3420
AAGGATCTTCACCTAGATCCTTTTAAATTAAAAATGAAGTTTTAA 3465
ATCAATCTAAAGTATATATGAGTAAACTTGGTCTGACAGTTACCA 3510
ATGCTTAATCAGTGAGGCACCTATCTCAGCGATCTGTCTATTTCG 3555
TTCATCCATAGTTGCCTGACTCCCCGTCGTGTAGATAACTACGAT 3600
ACGGGAGGGCTTACCATCTGGCCCCAGTGCTGCAATGATACCGCG 3645
AGACCCACGCTCACCGGCTCCAGATTTATCAGCAATAAACCAGCC 3690
AGCCGGAAGGGCCGAGCGCAGAAGTGGTCCTGCAACTTTATCCGC 3735
CTCCATCCAGTCTATTAATTGTTGCCGGGAAGCTAGAGTAAGTAG 3780
TTCGCCAGTTAATAGTTTGCGCAACGTTGTTGCCATTGCTGCAGG 3825
CATCGTGGTGTCACGCTCGTCGTTTGGTATGGCTTCATTCAGCTC 3870
CGGTTCCCAACGATCAAGGCGAGTTACATGATCCCCCATGTTGTG 3915
CAAAAAAGCGGTTAGCTCCTTCGGTCCTCCGATCGTTGTCAGAAG 3960
TAAGTTGGCCGCAGTGTTATCACTCATGGTTATGGCAGCACTGCA 4005
TAATTCTCTTACTGTCATGCCATCCGTAAGATGCTTTTCTGTGAC 4050
TGGTGAGTACTCAACCAAGTCATTCTGAGAATAGTGTATGCGGCG 4095
ACCGAGTTGCTCTTGCCCGGCGTCAACACGGGATAATACCGCGCC 4140
ACATAGCAGAACTTTAAAAGTGCTCATCATTGGAAAACGTTCTTC 4185
GGGGCGAAAACTCTCAAGGATCTTACCGCTGTTGAGATCCAGTTC 4230
GATGTAACCCACTCGTGCACCCAACTGATCTTCAGCATCTTTTAC 4275
TTTCACCAGCGTTTCTGGGTGAGCAAAAACAGGAAGGCAAAATGC 4320
```

```
CGCAAAAAAGGGAATAAGGGCGACACGGAAATGTTGAATACTCAT    4365
ACTCTTCCTTTTTCAATATTATTGAAGCATTTATCAGGGTTATTG    4410
TCTCATGAGCGGATACATATTTGAATGTATTTAGAAAAATAAACA    4455
AATAGGGGTTCCGCGCACATTTCCCCGAAAAGTGCCACCTGACGT    4500
CTAAGAAACCATTATTATCATGACATTAACCTATAAAAATAGGCG    4545
TATCACGAGGCCCTTTCGTCTTCAAGAATTAGCTTGGCCATTGCA    4590
TACGTTGTATCCATATCATAATATGTACATTTATATTGGCTCATG    4635
TCCAACATTACCGCCATGTTGACATTGATTATTGACTAGTTATTA    4680
ATAGTAATCAATTACGGGGTCATTAGTTCATAGCCCATATATGGA    4725
GTTCCGCGTTACATAACTTACGGTAAATGGCCCGCCTGGCTGACC    4770
GCCCAACGACCCCCGCCCATTGACGTCAATAATGACGTATGTTCC    4815
CATAGTAACGCCAATAGGGACTTTCCATTGACGTCAATGGGTGGA    4860
GTATTTACGGTAAACTGCCCACTTGGCAGTACATCAAGTGTATCA    4905
TATGCCAAGTACGCCCCCTATTGACGTCAATGACGGTAAATGGCC    4950
CGCCTGGCATTATGCCCAGTACATGACCTTATGGGACTTTCCTAC    4995
TTGGCAGTACATCTACGTATTAGTCATCGCTATTACCATGGTGAT    5040
GCGGTTTTGGCAGTACATCAATGGGCGTGGATAGCGGTTTGACTC    5085
ACGGGGATTTCCAAGTCTCCACCCCATTGACGTCAATGGGAGTTT    5130
GTTTTGGCACCAAAATCAACGGGACTTTCCAAAATGTCGTAACAA    5175
CTCCGCCCCATTGACGCAAATGGGCGGTAGGCGTGTACGGTGGGA    5220
GGTCTATATAAGCAGAGCTCAATAAAAGAGCCCACAACCCCTCAC    5265
TCGGCGCGCCAGTCTTCCGATAGACTGCGTCGCCCGGGTACCCGT    5310
ATTCCCAATAAAGCCTCTTGCTGTTTGCATCCGAATCGTGGTCTC    5355
GCTGTTCCTTGGGAGGGTCTCCTCTGAGTGATTGACTACCCACGA    5400
CGGGGGTCTTTCATTTGGGGGCTCGTCCGGGATTTGGAGACCCCT    5445
GCCCAGGGACCACCGACCCACCACCGGGAGGTAAGCTGGCCAGCA    5490
ACTTATCTGTGTCTGTCCGATTGTCTAGTGTCTATGTTTGATGTT    5535
ATGCGCCTGCGTCTGTACTAGTTAGCTAACTAGCTCTGTATCTGG    5580
CGGACCCGTGGTGGAACTGACGAGTTCTGAACACCCGGCCGCAAC    5625
CCTGGGAGACGTCCCAGGGACTTTGGGGGCCGTTTTTGTGGCCCG    5670
ACCTGAGGAAGGGAGTCGATGTGGAATCCGACCCCGTCAGGATAT    5715
GTGGTTCTGGTAGGAGACGAGAACCTAAAACAGTTCCCGCCTCCG    5760
TCTGAATTTTTGCTTTCGGTTTGGAACCGAAGCCGCGCGTCTTGT    5805
CTGCTGCAGCGCTGCAGCATCGTTCTGTGTTGTCTCTGTCTGACT    5850
GTGTTTCTGTATTTGTCTGAAAATTAGGGCCAGACTGTTACCACT    5895
CCCTTAAGTTTGACCTTAGGTCACTGGAAAGATGTCGAGCGGATC    5940
GCTCACAACCAGTCGGTAGATGTCAAGAAGAGACGTTGGGTTACC    5985
TTCTGCTCTGCAGAATGGCCAACCTTTAACGTCGGATGGCCGCGA    6030
GACGGCACCTTTAACCGAGACCTCATCACCCAGGTTAAGATCAAG    6075
GTCTTTTCACCTGGCCCGCATGGACACCCAGACCAGGTCCCCTAC    6120
ATCGTGACCTGGGAAGCCTTGGCTTTTGACCCCCCTCCCTGGGTC    6165
AAGCCCTTTGTACACCCTAAGCCTCCGCCTCCTCTTCCTCCATCC    6210
GCCCCGTCTCTCCCCCTTGAACCTCCTCGTTCGACCCCGCCTCGA    6255
TCCTCCCTTTATCCAGCCCTCACTCCTTCTCTAGGCGCCGGAATT    6300
GAAGATCTGGGCAGGAAGAGGGCCTATTTCCCATGATTCCTTCAT    6345
ATTTGCATATACGATACAAGGCTGTTAGAGAGATAATTAGAATTA    6390
ATTTGACTGTAAACACAAAGATATTAGTACAAAATACGTGACGTA    6435
GAAAGTAATAATTTCTTGGGTAGTTTGCAGTTTTAAAATTATGTT    6480
```

TTAAAATGGACTATCATATGCTTACCGTAACTTGAAAGTATTTCG 6525
ATTTCTTGGCTTTATATATCTTGTGGAAAGGACGAGG 6566
