# Supplementary material for: In-Vitro Subtype-Specific Modulation of HIV-1 Trans-Activator of Transcription (Tat) on RNAi Silencing Suppressor Activity and Cell Death
Source: Viruses. 2019 Oct 23;11(11):976. doi: 10.3390/v11110976 (PMC6893708; doi:10.3390/v11110976)
Supplement: Supplementary file 1 [file viruses-11-00976-s001.zip › S5.pdf]

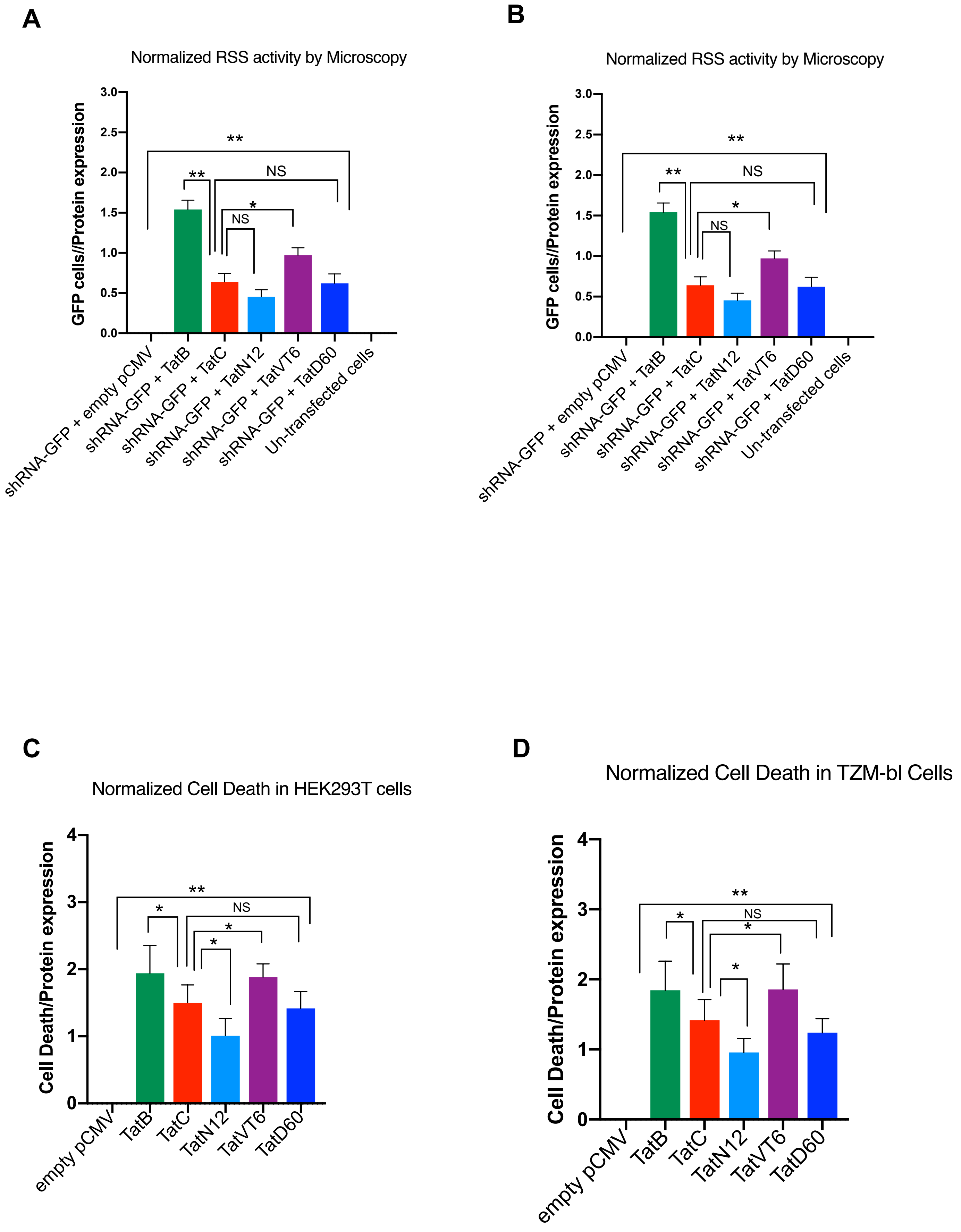
A
Normalized RSS activity by Microscopy
GFP cells//Protein expression
3.0
2.5
2.0
1.5
1.0
0.5
0.0
**
**
NS
*
NS
shRNA-GFP + empty pCMV
shRNA-GFP + TatB
shRNA-GFP + TatC
shRNA-GFP + TatN12
shRNA-GFP + TatVT6
shRNA-GFP + TatD60
Un-transfected cells
B
Normalized RSS activity by Microscopy
GFP cells//Protein expression
3.0
2.5
2.0
1.5
1.0
0.5
0.0
**
**
NS
*
NS
shRNA-GFP + empty pCMV
shRNA-GFP + TatB
shRNA-GFP + TatC
shRNA-GFP + TatN12
shRNA-GFP + TatVT6
shRNA-GFP + TatD60
Un-transfected cells
C
Normalized Cell Death in HEK293T cells
Cell Death/Protein expression
4
3
2
1
0
**
*
NS
*
*
empty pCMV
TatB
TatC
TatN12
TatVT6
TatD60
D
Normalized Cell Death in TZM-bl Cells
Cell Death/Protein expression
4
3
2
1
0
**
*
NS
*
*
empty pCMV
TatB
TatC
TatN12
TatVT6
TatD60
