# Supplementary figures and images for: In-Vitro Subtype-Specific Modulation of HIV-1 Trans-Activator of Transcription (Tat) on RNAi Silencing Suppressor Activity and Cell Death
Source: Viruses. 2019 Oct 23;11(11):976. doi: 10.3390/v11110976 (PMC6893708; doi:10.3390/v11110976)

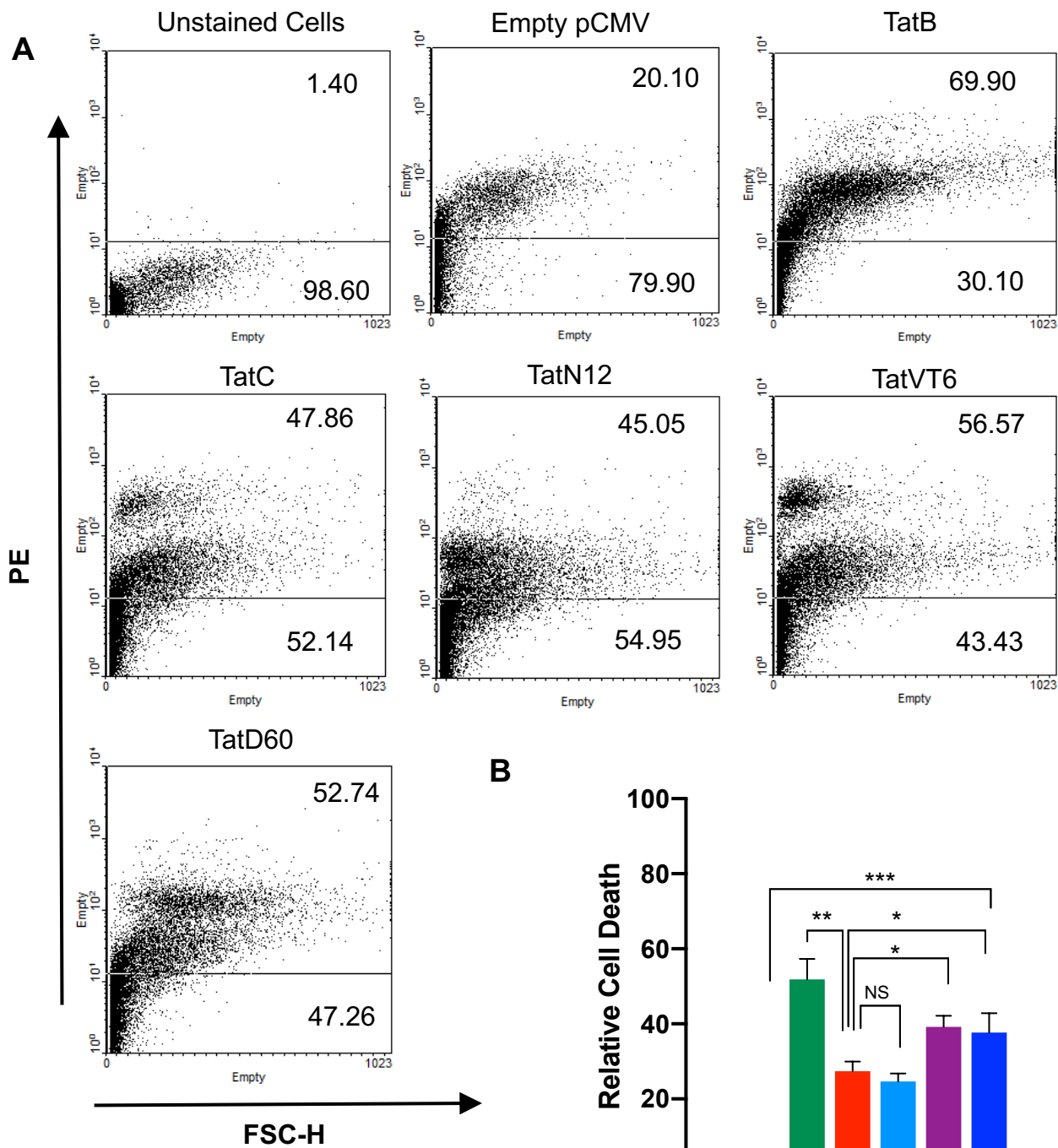

**B**

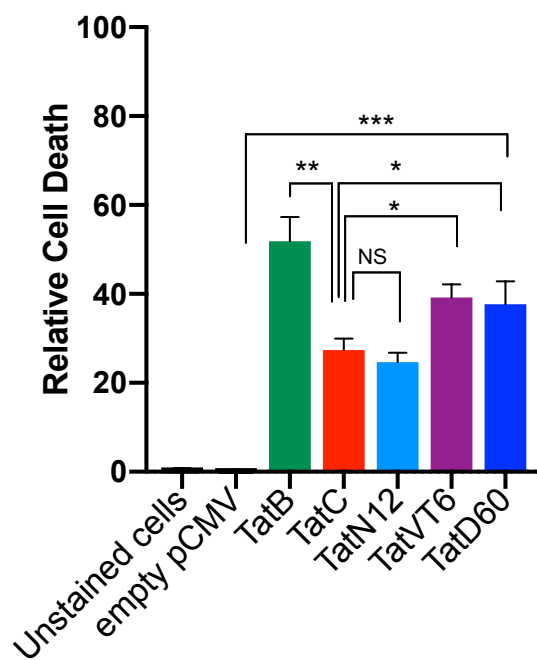

Supplement: Supplementary file 1 [file viruses-11-00976-s001.zip › S2.pdf]

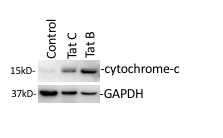

Supplement: Supplementary file 1 [file viruses-11-00976-s001.zip › S3.tiff]

Un-transfected cells

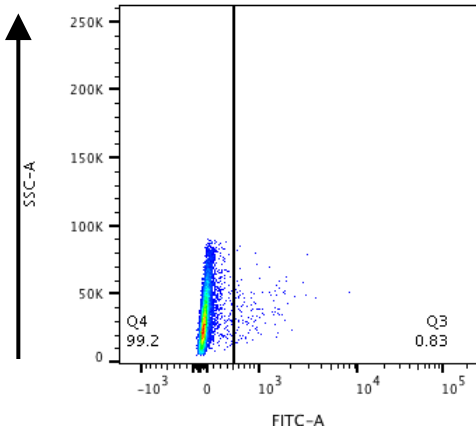

GFP cells + TatB

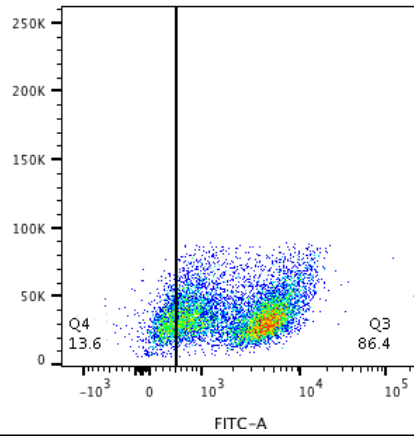

GFP cells + TatC

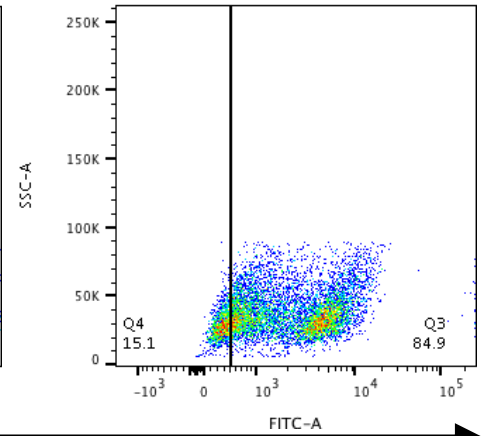

Supplement: Supplementary file 1 [file viruses-11-00976-s001.zip › S4.pdf]
